# MicroRNA expression profile of HCT-8 cells in the early phase of *Cryptosporidium parvum* infection

**DOI:** 10.1186/s12864-018-5410-6

**Published:** 2019-01-14

**Authors:** Chenrong Wang, Limin Liu, Huili Zhu, Lu Zhang, Rongjun Wang, Zhenjie Zhang, Jianying Huang, Sumei Zhang, Fuchun Jian, Changshen Ning, Longxian Zhang

**Affiliations:** 1 College of Animal Science and Veterinary Medicine, Henan Agricultural University, Zhengzhou, 450002 People’s Republic of China; 2 International Joint Research Laboratory for Zoonotic Diseases of Henan, Zhengzhou, 450002 People’s Republic of China; 3 College of Animal Science and Veterinary Medicine, Henan Institute of Science and Technology, Xinxiang, 453003 People’s Republic of China

**Keywords:** *Cryptosporidium parvum*, Intestinal epithelium, Infection, MicroRNA

## Abstract

**Background:**

*Cryptosporidium parvum* is an important zoonotic parasitic disease worldwide, but the molecular mechanisms of the host–parasite interaction are not fully understood. Noncoding microRNAs (miRNAs) are considered key regulators of parasitic diseases. Therefore, we used microarray, qPCR, and bioinformatic analyses to investigate the intestinal epithelial miRNA expression profile after *Cryptosporidium parvum* infection.

**Results:**

Twenty miRNAs were differentially expressed after infection (four upregulated and 16 downregulated). Further analysis of the differentially expressed miRNAs revealed that many important cellular responses were triggered by *Cryptosporidium parvum* infection, including cell apoptosis and the inflammatory and immune responses.

**Conclusions:**

This study demonstrates for the first time that the miRNA expression profile of human intestinal epithelium cells is altered by *C. parvum* infection. This dysregulation of miRNA expression may contribute to the regulation of host biological processes in response to *C. parvum* infection, including cell apoptosis and the immune responses. These results provide new insight into the regulatory mechanisms of host miRNAs during cryptosporidiosis, which may offer potential targets for future *C. parvum* control strategies.

**Electronic supplementary material:**

The online version of this article (10.1186/s12864-018-5410-6) contains supplementary material, which is available to authorized users.

## Background

*Cryptosporidium* is a genus of protozoan parasites that infect the gastrointestinal epithelium and other mucosal surfaces of their hosts, which include humans and domestic and wild animals worldwide [1–3]. *Cryptosporidium* is a major cause of moderate to severe diarrhea in children younger than 2 years, particularly infants, and is second only to rotavirus in this regard [4]. Morbidity and mortality are associated with cryptosporidial infections in patients with acquired immunodeficiency syndrome (AIDS) and young children [1, 5]. *Cryptosporidium parvum* is one of the species most commonly involved in human cryptosporidial infections [1]. Despite intensive efforts over the past 30 years, the precise molecular pathogenic mechanisms of cryptosporidial infection are not fully understood.

MicroRNAs (miRNAs) are endogenous noncoding small RNAs (22 nucleotides) that regulate gene expression at the posttranscriptional level and play important roles in the regulation of diverse pathological processes, including cell proliferation, differentiation, metabolism, the immune response, and apoptosis [6–8]. The miRNAs in mammalian cells have been shown to play crucial roles in the cellular responses to infection by diverse pathogens, including viruses, parasites, and bacteria [9–11]. Increasing evidence suggests that host miRNAs help the host to clear parasites by regulating the toll-like receptor 4 (TLR4) signaling pathways and the release of antimicrobial peptides [12, 13]. For example, *C. parvum* infection reduces the expression of the let-7 family miRNAs in biliary epithelial cells, which increases SNAP23 expression, coordinating the release of exosomes carrying antimicrobial-peptide in response to *C. parvum* infection [13]. The upregulation of miR-27b expression enhances the anti-*C. parvum* responses of biliary epithelial cells [14]. Some evidence suggests that host miRNAs are also used by *C. parvum* to enhance its own survival. For example, suppressor of cytokine signaling (SOCS) proteins are negative regulators of cytokine signaling [15]. *Cryptosporidium parvum* infection downregulates the expression of miR-98 and let-7 to induce SOCS protein expression in biliary epithelial cells [16]. The upregulation of miR-21 after *C. parvum* infection inhibits the activation of TLR4/NF-κB signaling in biliary epithelial cells by targeting PDCD4, a proinflammatory protein that promotes the activation of NF-κB [17]. Therefore, studying the changes in the host miRNA expression profile after *C. parvum* infection will extend our understanding of the interaction between *C. parvum* and its host.

There has been no report of the changes in the miRNA expression profile in the intestinal epithelium after *C. parvum* infection. Therefore, the objective of this study was to analyze the intestinal epithelial miRNA expression profile after *C. parvum* infection using microarray and bioinformatics analyses in an in vitro model using human ileocecal HCT-8 adenocarcinoma cells.

## Methods

### Cell and parasite

Human ileocecal adenocarcinoma (HCT-8) cells (American Type Culture Collection, Manassas, VA) were cultured and maintained in Dulbecco’s modified Eagle’s medium (DMEM) supplemented with 10% fetal bovine serum, 4 mmol/L l-glutamine, 100 U/mL penicillin, and 100 U/mL streptomycin at 37 °C in a humidified 5% CO_2_ incubator.

Neonatal calves were purchased from Ruiya Animal Husbandry Co., Ltd. (Zhengzhou, China). Oocysts of *C. parvum* (IId subtype) were maintained in infected neonatal calves, purified from their feces, and stored at 4 °C in 2.5% potassium dichromate until use. Before use, the oocysts were washed with phosphate-buffered saline (PBS), treated with 10% hypochlorite for 10 min at 4 °C, and washed again [18]. The infected calf was killed by anesthesia (113 mg sodium pentobarbital/kg, Intravenous injection) and carcass was treated harmlessly by a specialized agency of our college.

The infection assays have been reported previously [18]. Briefly, cell monolayers in six-well cell culture dishes were inoculated with 100 μL of purified oocysts (1 × 10^7^/well) in DMEM, that the oocyst:host cell ratio is 5:1. Heat-inactivated (100 °C, 5 min) oocysts were used as the controls. After 2.5 h, oocysts and sporozoites remaining in the supernatant were removed and replaced with DMEM containing 2% fetal bovine serum, 4 mmol/L l-glutamine, 100 U/mL penicillin, 100 g/mL streptomycin, 0.3 g/L bicarbonate, and 0.02 g/L taurocholate. The plates were incubated at 37 °C in a humidified 5% CO_2_ incubator.

### Total RNA isolation and quality control

At 4 and 12 h postinfection, the cells were washed three times with PBS, and 1 mL of TRIzol Reagent (Invitrogen, Carlsbad, CA, USA) was added to each well. The total RNA from three uninfected separate monolayers and three infected separate monolayers were isolated according to the manufacturer’s protocol.

The quality of the RNA was measured with a NanoDrop 2000 spectrophotometer (Thermo Fisher Scientific, Inc., Waltham, MA, USA). RNA purity was assessed from the absorbance (A) of the solutions at various wavelengths: 2.0 > A_260_/A_280_ > 1.8 and A_260_/A_230_ > 2.0 were considered to indicate high purity. RNA integrity was determined from the 28S:18S rRNA ratio determined with an electrophoretic analysis.

### Microarray analysis

miRNA microarrays based on miRBase v20.0 (http://www.mirbase.org/) were used to study the expression profiles of 2555 mature miRNAs. Microarray chips are synthesized by LC Sciences and have patent rights. The chips are made by a flexible, in situ synthesis method using conventional chemistry. The technology encompasses a novel high-throughput biopolymer synthesis chemistry carried out using a new class of microfluidic reaction devices and an advanced digital light synthesizer apparatus and is developed for carrying out picoliter scale chemical and/or biochemical reactions. Briefly, Hybridization was performed using the μParaflo® microfluidic array technology (LC Sciences, Hangzhou, China), according to the manual. The assay started from 4 to 8 μg total RNA sample were 3^′^-extended with a poly(A) tail using poly(A) polymerase. An oligonucleotide tag that binding to Cy3 dyes in later chip hybridization was then ligated to the poly(A) tail. Hybridization was performed overnight on a μParaflo microfluidic chip using a micro-circulation pump (Atactic Technologies). On the microfluidic chip, each detection probe consisted of a chemically modified nucleotide coding segment complementary to target microRNA (from miRBase, http://www.mirbase.org/). The detection probes were made by in situ synthesis using PGR (photogenerated reagent) chemistry. The hybridization melting temperatures were balanced by chemical modifications of the detection probes. Hybridization used 100 μL 6xSSPE buffer (0.90 M NaCl, 60 mM Na2HPO4, 6 mM EDTA, pH 6.8) containing 25% formamide at 34 °C. After RNA hybridization, oligonucleotide tag-conjugating Cy3 dye were circulated through the microfluidic chip for dye staining. Fluorescence images were collected using a laser scanner (GenePix 4000B, Molecular Device) and digitized using Array-Pro image analysis software (Media Cybernetics). Data were analyzed by first subtracting the background and then normalizing the signals using a LOWESS filter (Locally-weighted Regression).

Each miRNA has a fold change in the signals detected in infected cells relative to the signals detected in uninfected cells and the *P* value that calculated with Student’s *t* test. Differentially expressed miRNAs was selected based on a fold change of > 2 or < − 2 and a P value of ≤0.05.

### Validation of microarray data with quantitative PCR (qPCR) analysis

miRNAs were prepared with an All-in-One™ miRNA qRT–PCR Detection Kit (GeneCopoeia, Rockville, MD, USA), according to the manufacturer’s instructions. Briefly, the extracted RNA was reverse-transcribed in the presence of a poly(A) polymerase and an oligo-dT adaptor. Quantitative PCR was then performed with SYBR Green detection using a forward primer specific for the mature miRNA sequence and a universal adaptor reverse primer. The forward primers used for the qPCRs are shown in Table 1. Each RNA was run in triplicate wells with three biological replicates, including three uninfected separate monolayers and three infected separate monolayers. The cycling protocol was: 95 °C for 10 min, followed by 40 cycles of 95 °C for 10 s and 60 °C for 20 s, with a final extension at 72 °C for 20 s. When all cycles were finished, a melting-curve analysis was performed. The baseline and cycle threshold (Ct) values were automatically determined for all plates with the Roche LightCycler® 96 Real-Time PCR System. The miRNA expression levels in each sample were normalized to the expression of the housekeeping gene *U6* [19–21]. The real-time PCR results were analyzed and expressed as the relative Ct value using the 2^−ΔΔCt^ method [22]. The mean and standard deviation (M ± SD) was calculated from three independent biological trials. Differences between infected and control group were measured using the t-test by SPSS 17.0 with *P* < 0.05 indicating significance.

**Table 1:** Specific primers used for qPCR

miRNAs	Forward primer
hsa-miR-122-5p	TGGAGTGTGACGATGGTGTTTG
hsa-miR-3591-3p	AGCATCACCATTGTCACACTCCAC
hsa-miR-5580-3p	CACAGTTGAAGAGAGCCAGCAC
hsa-miR-181d-3p	TCATAGGGGGCTCAATGTCAC
hsa-miR-454-5p	CGCCTATCGCTATTGTCTCTGC
hsa-let-7b-3p	GCATACAACCTACTGCCTTCCC
U6	TGGCAAGGATGACACGCAAAT

### Predicted target genes of differentially expressed miRNAs, gene ontology (GO) and Kyoto encyclopedia of genes and genomes

#### (KEGG) pathway analyses

Despite an abundance of online software for predicting human microRNA targets, many human miRNAs are only included in TargetScanHuman7.1 and miRDB. Therefore, the target genes of the miRNAs were predicted with the two online programs TargetScanHuman7.1 (http://www.targetscan.or g/vert_71/) [23] and miRDB (http://www.mirdb.org/cgi-bin/ search.cgi) [24]. The target genes of the differentially expressed miRNAs were analyzed in terms of their GO categories and KEGG pathways using the Database for Annotation, Visualization and Integrated Discovery (DAVID) (https://david.ncifcrf.gov/), a gene annotation tool [25].

#### miRNA–target gene network analysis

The miRNA- predicted target genes regulatory network was constructed with the miRNA-target gene pairs using the Cytoscape software(www.cytoscape.org) [26–28], based on the interactions of the miRNAs and genes [29], Briefly, a text file containing miRNAs and predicted target genes was imported in Cytoscape software and optimized the visual features of nodes and edges, such as shape and color. With an miRNA–target gene network, we can identify the crucial miRNA which have the highest connectivity to other miRNAs. The higher the connectivity of a miRNA was, the more important it was in the network. Also, we can find the key genes which were the common target genes of the differentially expressed miRNAs.

## Results

### Differentially expressed miRNAs

**Fig. 1:**
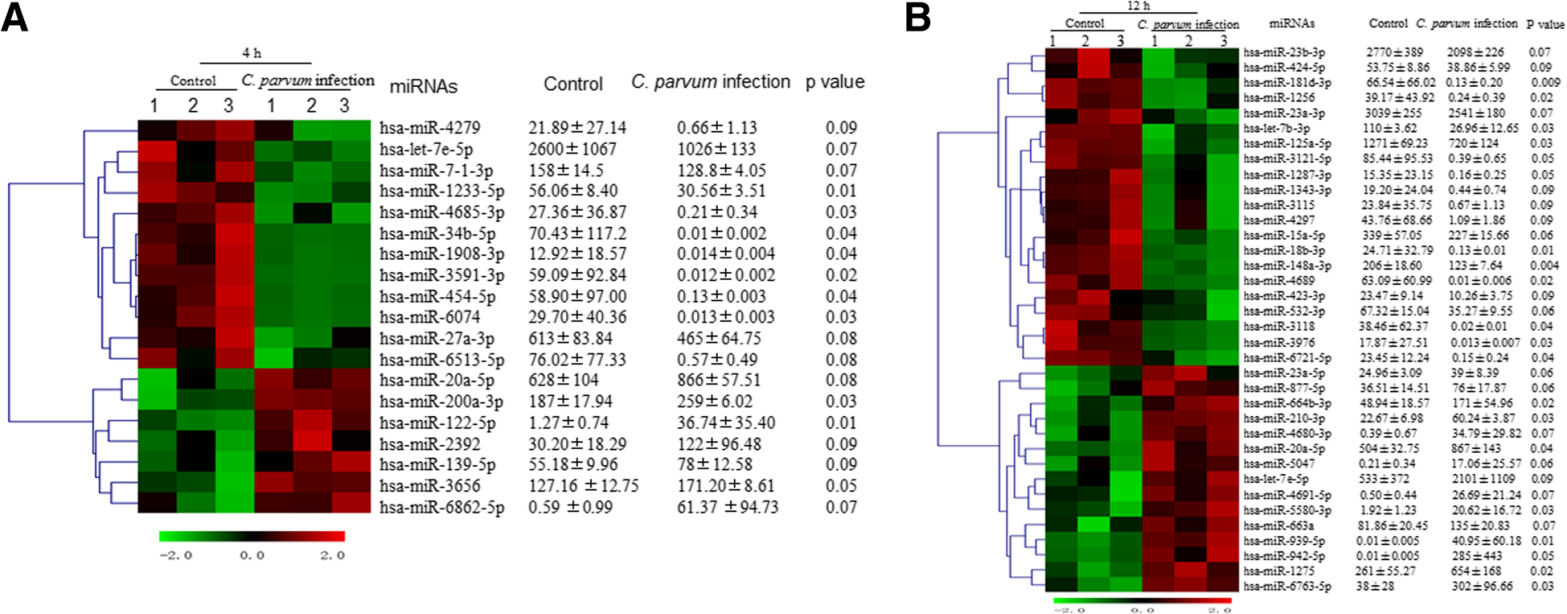
Expression profiling of miRNAs in HCT-8 cells after *C. parvum* infection. Expression profiles of miRNAs in HCT-8 cells at 4 h after *C. parvum* infection. The horizontal axis indicates samples of non-infected cells (*n* = 3; Control 1, 2, and 3) and cells after exposure to live *C. parvum* for 12 h (n = 3, *C. parvum* 1, 2, and 3) (**a**). Expression profiles of miRNAs in HCT-8 cells at 12 h after *C. parvum* infection. The horizontal axis indicates samples of non-infected cells (n = 3; Control 1, 2, and 3) and cells after exposure to live *C. parvum* for 12 h (n = 3, *C. parvum* 1, 2, and 3) (**b**)

A total of 2555 mature human miRNA sequences were detected in *C. parvum*-infected and uninfected HCT-8 cells using miRNA microarrays (Sanger miRBase v20.0). A dendrogram produced with a hierarchical clustering analysis of the miRNAs differentially expressed in *C. parvum*-infected and -uninfected cells is shown in Fig. 1. In total, 20 miRNAs (four upregulated and 16 downregulated) were significantly differentially expressed in the infected HCT-8 cells compared to uninfected cells. These differentially expressed miRNAs (with fold changes ≥2 and *P* ≤ 0.05) are shown in Table 2 with *P* values. From this table, it is clear that downregulated miRNAs were more frequent than upregulated miRNAs (*P* < 0.05). Among these miRNAs, hsa-miR-942-5p was most strongly upregulated and hsa-miR-4689 was most strongly downregulated.

**Table 2:** Differentially expressed miRNAs in the infected HCT-8 cells compared to uninfected cells

Infection time	hsa-microRNAs	Log2 fold change	*P*-value
4 h	hsa-miR-122-5p	+ 4.85	0.01
	hsa-miR-3591-3p	−12.29	0.02
	hsa-miR-6074	−11.12	0.03
	hsa-miR-454-5p	−12.16	0.04
	hsa-miR-34b-5p	−12.19	0.04
	hsa-miR-4685-3p	−7.02	0.03
	hsa-miR-1908-3p	−9.88	0.04
12 h	hsa-miR-942-5p	+ 15.26	0.05
	hsa-miR-5580-3p	+ 3.42	0.03
	hsa-miR-6763-5p	+ 2.99	0.03
	hsa-miR-181d-3p	−8.99	0.009
	hsa-miR-18b-3p	−10.94	0.01
	hsa-miR-4689	−12.30	0.02
	hsa-miR-1256	−7.35	0.02
	hsa-miR-3976	−10.42	0.03
	hsa-let-7b-3p	−2.09	0.03
	hsa-miR-6721-5p	−7.29	0.04
	hsa-miR-3118	−11.23	0.04
	hsa-miR-3121-5p	−7.77	0.05
	hsa-miR-1287-3p	−6.62	0.05

* “+”represents up-regulation, “-”represents down-regulation

### Validation of miRNA microarray data with qPCR

**Fig. 2:**
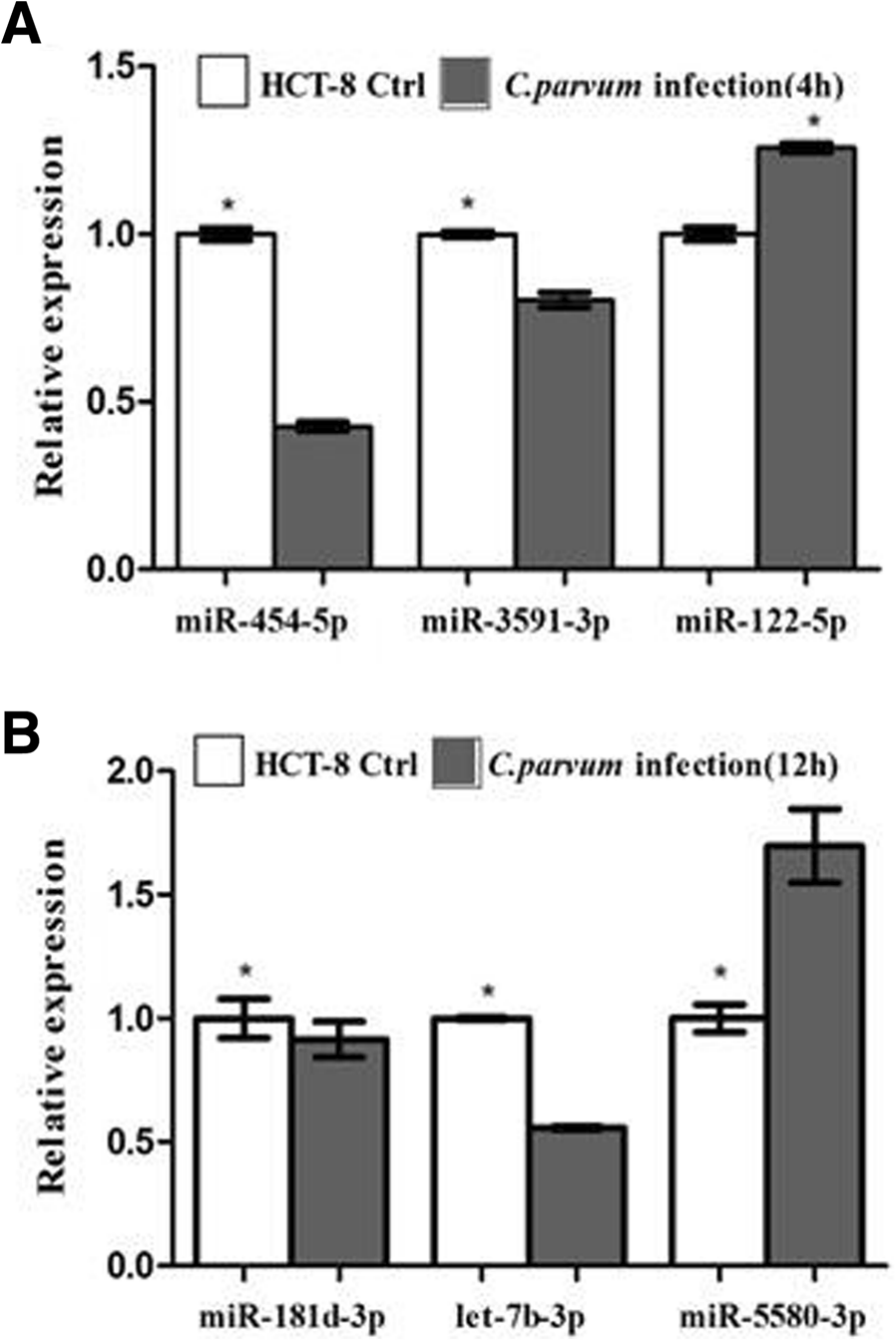
qPCR confirmation of the miRNA microarray data. qPCR confirmation of the miRNAs differentially expressed at 4 h after *C. parvum* infection (**a**). qPCR confirmation for the miRNAs differentially expressed at 12 h after *C. parvum* infection (**b**). Data are the means and standard errors of the means of triplicate experiments

Six selected miRNAs and the housekeeping U6 mRNA were assayed with qPCR to confirm the expression profiles identified with the microarray analysis. The expression patterns determined with qPCR agreed well with those determined from the microarray data (Fig. 2).

### Predicting the target genes of differentially expressed miRNAs

We predicted the target genes for all the miRNAs differentially expressed in HCT-8 cells after *C. parvum* infection. Target genes were predicted for only 18 of the 20 differentially expressed miRNAs using two different online software programs, TargetScan and miRDB.

### GO analysis of differentially expressed miRNAs

**Fig. 3:**
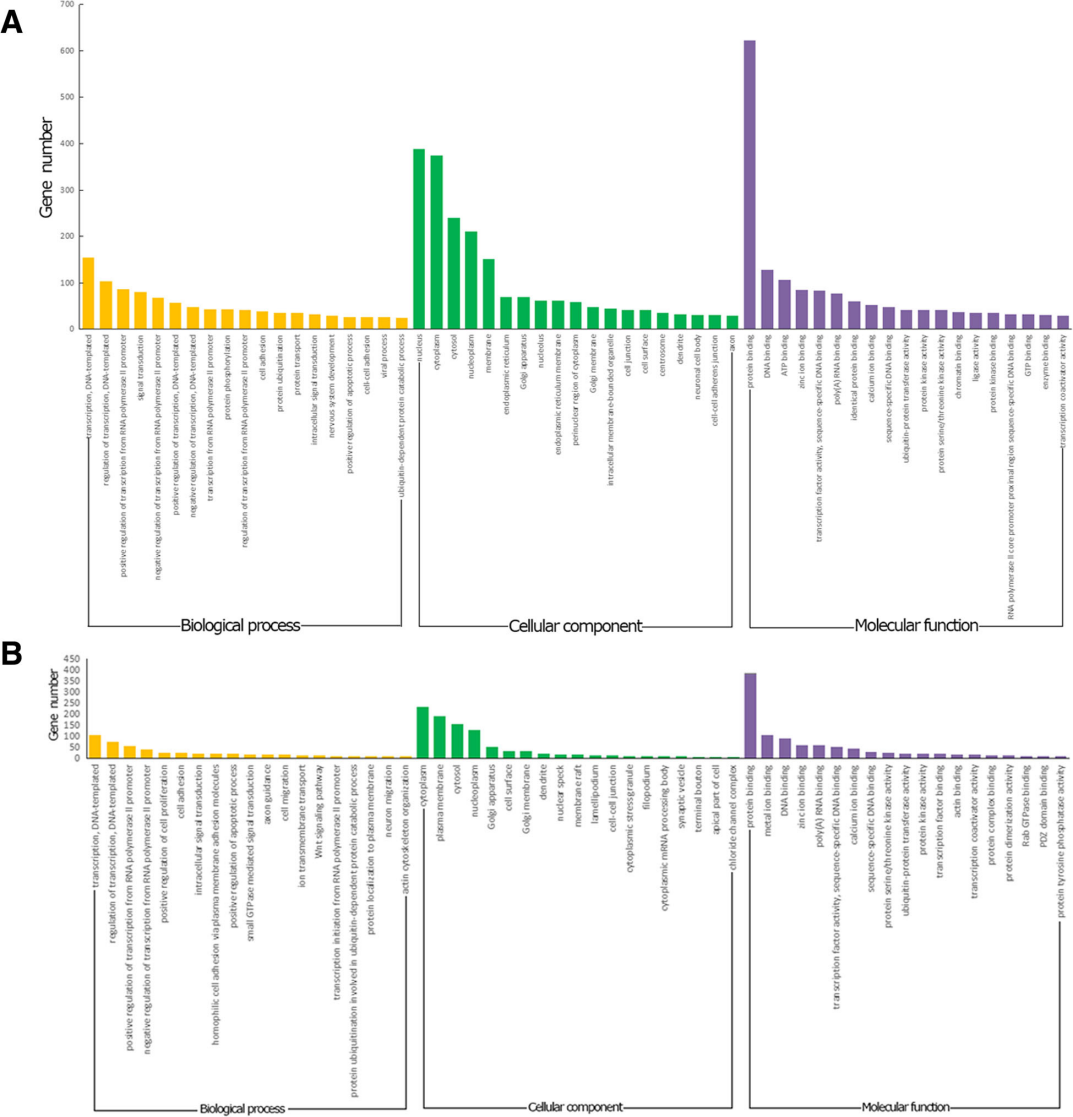
GO analysis of targets of differentially expressed miRNAs at 4 h and 12 h respectively. According to gene count, top 19 GO terms (*P* < 0.05) are shown. GO analysis of targets of differentially expressed miRNAs at 4 h (**a**). GO analysis of targets of differentially expressed miRNAs at 4 h (**b**)

To further understand the functions of the differentially expressed miRNAs, a GO analysis of the target genes was performed. DAVID gene annotation was applied to explain the biological effects of the differentially expressed miRNAs based on their target genes. The GO annotations of differentially expressed miRNAs (with *P* < 0.05) are shown in Fig. 3. The GO-specific functions mainly involved biological processes (e.g., transcription initiation from the RNA polymerase II promoter, apoptotic process, and immune response), cell components (e.g., membrane, actin cytoskeleton, focal adhesion, plasma membrane, cell surface, and apical part of the cell), and molecular functions (e.g., protein binding, ATP binding, calcium ion binding). Among the GO annotations (with *P* < 0.05), hsa-miR-34b-5p, hsa-miR-942-5p, hsa-miR-3591-3p, hsa-miR-18b-3p and hsa-miR-3976 were involved in the regulation of apoptotic or autophagy processes, hsa-miR-34b-5p and hsa-miR-3591-3p were involved in the regulation of the immune response (Additional file 1: Figure S1). Thus, the GO analysis of the differentially expressed miRNA target genes revealed that some have potentially important biological functions in regulating the host against *C. parvum* infection.

### KEGG pathway analysis of differentially expressed miRNAs

The main KEGG pathway annotations of the target genes (with P < 0.05) are shown in Table 3. Among these KEGG pathways, several were associated with the immune response (e.g., cytokine receptor interaction, chemokine signaling pathway, and inflammatory mediator regulation of TRP channels), apoptosis (e.g., MAPK signaling pathway and ERBB signaling pathway), cytoskeleton (e.g., actin cytoskeleton and WNT signaling pathway), and cell adhesion (e.g., cell adhesion molecules).

**Table 3:** KEGG pathways of differentially expressed miRNAs

Hsa-miRNA	Pathway	Gene	*P* value
miR-34b-5p	Ubiquitin mediated proteolysis	CBLB, CBL, NHLRC1, BTRC, NEDD4, PIAS1	0.0073
	AMPK signaling pathway	ELAVL1, RAB10, RAB14, CREB1, HNF4, APRKA2	0.04
	Notch signaling pathway	APH1A, DLL1, JAG1, NOTCH1, NOTCH2	0.017
	Hippo signaling pathway	MOB1B, WNT2B, BTRC, BMPR2, DLG1, GSK3B	0.013
miR-942-5p	Ras signaling pathway	RAF1, TEK, CHUK, FGF9MAPK10, MRAS…	0.006
	ErbB signaling pathway	RAF1, CDKN1B, MAPK10, NRAS, PAK2, PIK3CD	0.0082
	MAPK signaling pathway	RAF1, TAOK1, TAB2, CACNA1E, CHUK,…	0.014
	Wnt signaling pathway	CREBBP, FBXW11, WNT9B, CCND1…	0.022
	p53 signaling pathway	CHEK1, CCND1, RFWD2, SERPINE1, ZMAT3	0.046
	PI3K-Akt signaling pathway	MCL1, RAF1, TEK, COL4A5, CHUK, CCND1…	0.047
miR-3591-3	Ubiquitin mediated proteolysis	CBLHERC3, CDC27, CUL5, MAP3K1, RFWD2...	0.0049
	ErbB signaling pathway	SOS2, CAMK2A, MAP2K4, NRG3, STAT5B	0.016
	MAPK signaling pathway	ELK4, SOS2, TAB2, DAXX, FGF14, MAP2K3…	0.017
miR-6721-5p	Cell adhesion molecules (CAMs)	CD22, CD276, CD40LG, CD8A, CADM3, CLDN19.	0.0018
	ErbB signaling pathway	CDKN1A, AKT2, ELK1, SRC, CAMK2A…	0.0037
	Ras signaling pathway	ABL1, AKT2, BCL2L1, ELK1, GNB2, RASA4B…	0.0087
	cGMP-PKG signaling pathway	AKT2, ATP1A2, ATP2B2, GNA11, GNAI2…	0.018
Let-7b-3p	Chemokine signaling pathway	AKT2, CX3CR1, CRKL, CRK, GNAI3, GNB4…	0.044
	actin cytoskeleton	CDC42RKL, GNA13, ROCK1, ROCK2, SOS2…	0.0086
	MAPK signaling pathway	CDC42, AKT2, CRKL, CRK, MECOM, RAP1B…	0.00085
	AMPK signaling pathway	HMGCR, PFKFB3, AKT2, ELAVL1, RAB10…	0.018
	Notch signaling pathway	DLL1, DLL4, HES1, JAG1, JAG2, MAML2, RBPJ…	0.031
	Wnt signaling pathway	LRP6, ROCK2, WNT5B, CTNNB1, CUL1…	0.0085
	TGF-beta signaling pathway	ROCK1, SP1, ACVR1, ACVR2B, BMPR2, CUL1...	0.0085
	cGMP-PKG signaling pathway	AKT2, ATP2B1, GNA13, GNAI3, ROCK1…	0.0036
miR-454-5p	Central carbon metabolism	AKT3, MAPK1, PDGFRA	0.012
	Rap1 signaling pathway	AKT3, MAPK1, PDGFRA, TLN2	0.016
miR-1287-3p	Ras signaling pathway	ABL2, BCL2L1, RAB5A, EFNA3, FGF12	0.021
miR-3121-5p	Cell adhesion molecules	CLDN2, HLA-C, NRXN1, PTPRF, SELE, SDC1	0.017
	Cytokine receptor interaction	CXCL14, KIT, RELT, TNFRSF21, ACVR1…	0.034
miR-1256	ErbB signaling pathway	KRAS, EGF, PIK3CG, TGFA	0.014
	Ras signaling pathway	KRAS, RAP1B, EGF, PIK3, CGRAC1, STK4	0.0093
	MAPK signaling pathwa	KRAS, MKNK2, RAP1B, TAB2, STK4…	8.50E-5
	Rap1 signaling pathway	KRAS, RAP1B, RAPGEF2, EGF, PIK3CG, RAC1	0.0068
miR-4685-3p	Inflammatory mediator regulation of TRP channels	ASIC1, ADCY3, ITPR2, MAP2K6	0.017
miR-5580-3p	ECM-receptor interaction	COL5A2, COL11A1, ITGA2, ITGA4, ITGA9	0.049

*“*P*-value” refers to significance of function enrichment

### Regulatory network of miRNAs and target genes

**Fig. 4:**
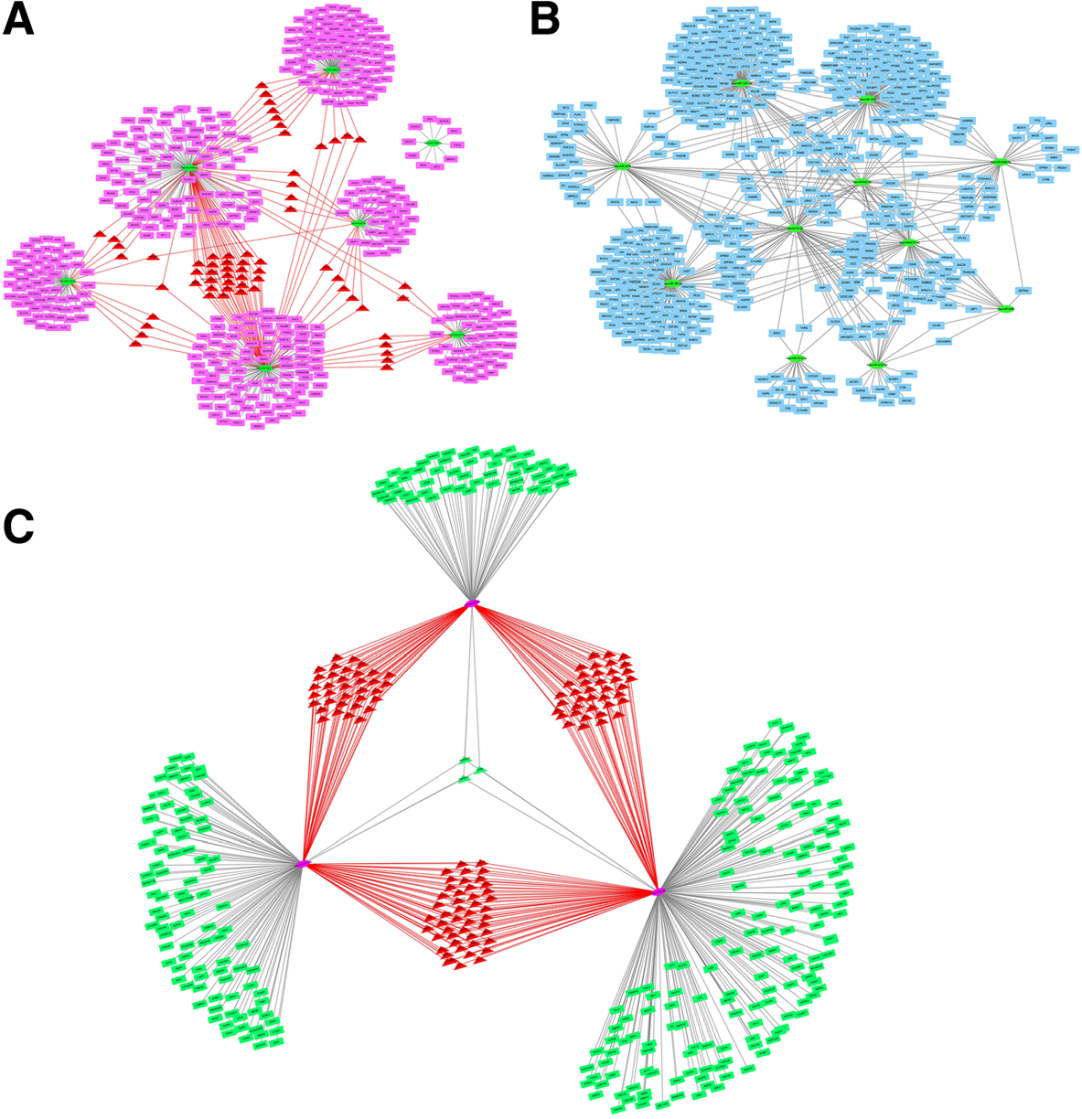
miRNA–target gene network. Network of miRNAs and their target genes at 4 h after *C. parvum* infection (**a**). Network of miRNAs and target genes at 12 h after *C. parvum* infection (**b**). Network of the key miRNAs and target genes at 12 h after *C. parvum* infection (**c**). In the diagram of the miRNA–gene network, the ellipses represent the miRNAs, the rectangles represent the target genes, and triangles represent the key target genes, with the interaction between them are represented by lines. The crucial miRNAs has the highest connectivity which refers to the number of edges possessed by one miRNA. The key genes are the common target genes of differentially expressed miRNAs

To investigate the interactions between the miRNAs and their target genes, we performed an miRNA–target gene network analysis (Fig. 4). From the network of the miRNA and their target genes at 4 h after *C. parvum* infection (Fig. 4a), we found among seven differentially expressed miRNAs at 4 h hsa-miR-34b-5p and hsa-miR-3591-3p have a connection with six differentially expressed miRNAs. Among eleven differentially expressed miRNAs at 12 h, hsa-miR-942-5p, hsa-let-7b-3p and hsa-miR-6721-5p have a connection with ten differentially expressed miRNAs(Fig. 4b). According to these datasets, the key miRNAs among the differentially expressed miRNAs were hsa-miR-34b-5p, hsa-miR-3591-3p, hsa-miR-942-5p, hsa-let-7b-3p, and hsa-miR-6721-5p Their common target genes of the key miRNAs at 12 h were shown in Fig. 4c.

## Discussion

Increasing evidence suggests that miRNAs regulate gene expression at the posttranscriptional level and thus affect a variety of biological processes, including the pathogenesis and progression of various diseases [30]. miRNAs also play important roles in the regulation of complex parasite–host interactions [10, 12, 14, 15]. *Cryptosporidium parvum*-induced miRNA expression in human biliary epithelial cells has already been investigated and analyzed [10]. However, there has been no report of the miRNA expression profile of human intestinal epithelial cells infected with *C. parvum*. Therefore, we analyzed the changes in the miRNA the profile of HCT-8 cells at 4 h and 12 h after *C. parvum* infection using microarray technology. Our results show that most miRNAs were not significantly differentially expressed in the infected HCT-8 cells compared to uninfected cells. However, some differentially expressed miRNAs were detected (four upregulated and 16 downregulated), implying that *C. parvum* infection alters the miRNA profile of intestinal epithelial cells. However, the differentially expressed miRNAs in the infected HCT-8 cells differed from those in biliary epithelial cells [10]. The changes in these specific miRNAs induced by *C. parvum* might be involved in the regulation of human intestinal epithelial cells in response to *C. parvum* infection. This is the first report to describe the alterations in miRNA expression induced by *C. parvum* infection in human intestinal epithelial cells, and extends our understanding of the interplay between *C. parvum* and its host at the miRNA level. The identification of the functions of these miRNA sheds new light on the molecular mechanisms triggered by *C. parvum* infection. Therefore, in this study, GO and KEGG analyses were used to identify the functions of the predicted target genes involved in *C. parvum* infection. Cell apoptosis is one of the ancient antiparasite strategies used by a host after its invasion by a parasite [31–34]. Research has shown that the early phase of *C. parvum* infection results in the apoptosis of HCT-8 cells [35]. However, miRNA-mediated cell apoptosis after *C. parvum* infection has not been reported. Our results suggest that the functions of hsa-miR-34b-5p, hsa-miR-942-5p, hsa-miR-18b-3p, hsa-miR-3976 and hsa-miR-3591-3p, most of which (not hsa-miR-942-5p) were downregulated after *C. parvum* infection, regulate apoptotic processes. Several reports have shown that hsa-miR-34b, hsa-miR-18a, and hsa-miR-942 regulate cell apoptosis in response to microbial infection. For example, upregulated hsa-miR-18a in macrophages after *Toxoplasma gondii* infection leads to the survival of the infected macrophages by targeting BIM [36]. Upregulated hsa-miR-30c-1 in human macrophages is involved in an anti-apoptosis response to *T. gondii* infection [37]. The downregulation of hsa-miR-34a induces cell apoptosis in *Influenza A virus*-infected A549 cells [38], and the downregulation of hsa-miR-942 enhances the apoptosis of HLCZ01 cells in response to *Hepatitis C virus* infection [39].

Other host antiparasite strategies are the immune responses [40–42]. As well as apoptosis, we found that hsa-miR-3591-3p and hsa-miR-34b-5p regulate the epithelial immune responses. Several reports have shown that hsa-miR-34a regulate host immune responses in response to microbial infection. For example, hsa-miR-34a negatively regulate the innate immune response to viral infections by targeting interferon β [43]. hsa-miR-3591-3p could increases interleukin 6 mRNA levels in THP-1 cells in response to lipopolysaccharide stimulation [44]. These data suggest that hsa-miR-3591-3p, hsa-miR-34b-5p, hsa-miR-942-5p, hsa-miR-18b-3p and hsa-miR-3976 may be involved in cell apoptosis or the immune responses to *C. parvum* infection. Hsa-miR-34b-5p, hsa-miR-3591-3p, hsa-miR-942-5p, hsa-let-7b-3p, and hsa-miR-6721-5p were also identified as the key miRNAs among the miRNAs differentially expressed after *C. parvum* infection. Therefore, the functions of hsa-let-7b-3p, hsa-miR-3591-3p, hsa-miR-34b-5p, hsa-miR-942-5p, hsa-miR-6721-5p, hsa-miR-18b-3p, hsa-miR-3976, and hsa-miR-3121-5p during *C. parvum* infection require further investigation.

There were some limitations to this study that should be addressed. First, the time points in the early phase of *C. parvum* infection was relatively limited and microRNA expression profile of HCT-8 cells in the later phase of *C. parvum* infection should be further analyzed to better understand the interplay between *C. parvum* and its host at the miRNA level. Second, we only assessed microRNA regulation in a single cell line, so the results should be confirmed with primary intestinal epithelial cells or another human intestinal epithelial cells line. Last, we should decrease the dose of oocysts used to infect the monolayer, because the high dose of oocysts may potentially have triggered a stress response in the host cell, the differentially regulated miRNA could reflect such a response and be unrelated to the actual *C. parvum* infection.

## Conclusions

In this study, we determined the miRNA expression profile of human intestinal epithelial cells infected by *C. parvum* for the first time, and demonstrated that the dysregulation of these miRNAs may regulate the host’s biological processes in response to *C. parvum* infection, including cell apoptosis and the immune responses. These findings extend our understanding of the miRNA regulatory network that operates during the host–*C. parvum* interaction. They also provide clues for further research into the regulatory mechanisms of miRNAs in human intestinal epithelial cells in response to *C. parvum* infection, which may identify potential targets for future *C. parvum* control strategies.

## Additional file

Additional file 1: **Figure S1.** GO analysis of targets of differentially expressed miRNAs involved in the regulation of apoptotic processes and the immune response. (PDF 521 kb)

## Abbreviations

DAVID: Database for annotation, visualization and integrated discovery
DMEM: Dulbecco’s modified Eagle’s medium
GO: Gene Ontology
KEGG: Kyoto encyclopedia of genes and genomes
SOCS: Suppressor of cytokine signaling
