# Supplementary figures and images for: MicroRNA expression profile of HCT-8 cells in the early phase of Cryptosporidium parvum infection
Source: BMC Genomics. 2019 Jan 14;20:37. doi: 10.1186/s12864-018-5410-6 (PMC6332841; doi:10.1186/s12864-018-5410-6)

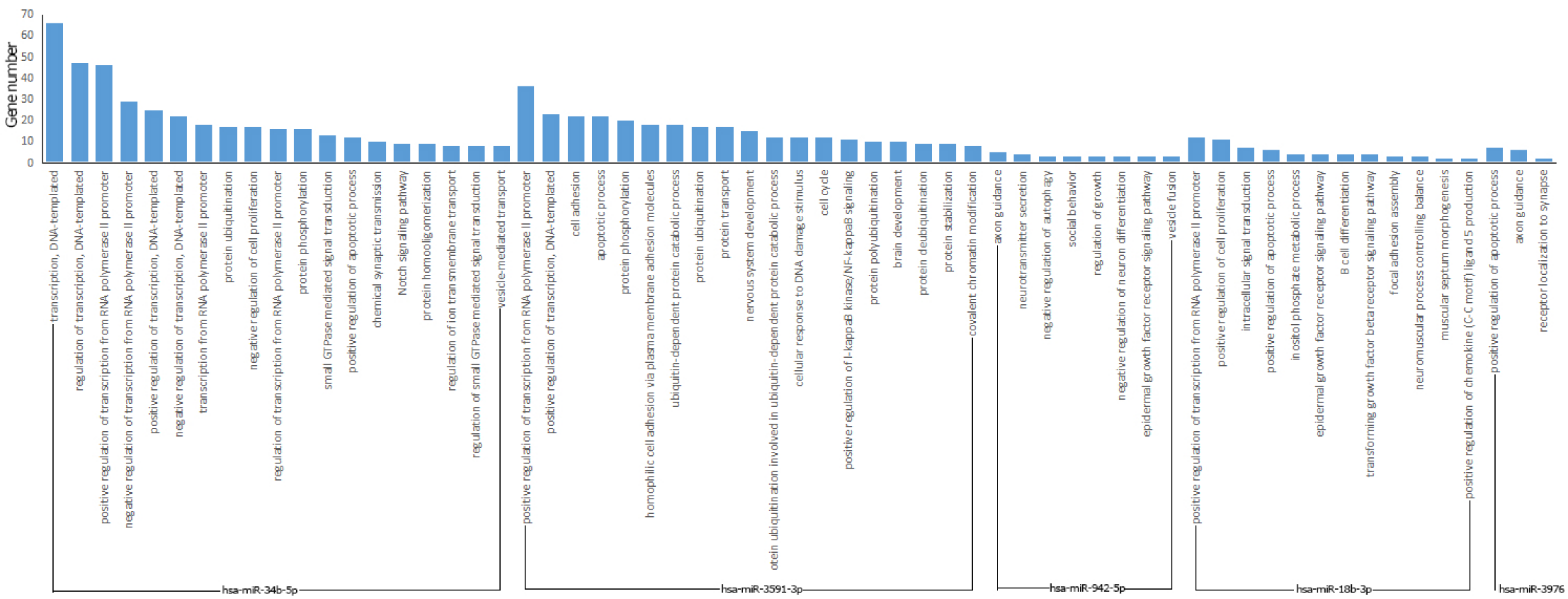

Supplement: Supplementary file 1 — Figure S1. GO analysis of targets of differentially expressed miRNAs involved in the regulation of apoptotic processes and the immune response. (PDF 521 kb) [file 12864_2018_5410_MOESM1_ESM.pdf]
